# Integrating Psychiatric, Psychotherapeutic, and Nursing Care in Intranasal Esketamine for Treatment-Resistant Depression

**DOI:** 10.3390/jcm15041629

**Published:** 2026-02-20

**Authors:** Vassilis Martiadis, Fabiola Raffone, Serena Testa, Concetta Iaccarino, Paolo Giunnelli, Ada Orrico, Emilia Carbone, Salvatore Clemente, Carmine De Simone, Antonietta Massa, Clemente Purcaro, Azzurra Martini, Enrico Pessina, Carlo Ignazio Cattaneo

**Affiliations:** 1 Department of Mental Health, Asl Napoli 1 Centro, 80125 Naples, Italy; fabiolaraffone@gmail.com (F.R.); concetta.iaccarino@aslnapoli1centro.it (C.I.); emilia.carbone@aslnapoli1centro.it (E.C.); carmine.desimone@aslnapoli1centro.it (C.D.S.); clemente.purcaro@aslnapoli1centro.it (C.P.); 2 Department of Mental Health, Asl Cuneo 2, Bra, 12042 Cuneo, Italyenricopessina@hotmail.com (E.P.); 3 Department of Mental Health, Asl Biella, 13900 Biella, Italy; carloignazio.cattaneo@aslbi.piemonte.it

**Keywords:** esketamine, treatment-resistant depression, nursing care, psychotherapy, patient care team, real-world evidence, patient-centred care, implementation

## Abstract

**Background/Objectives**: Intranasal esketamine has emerged as an effective treatment for patients with treatment-resistant depression (TRD), providing rapid symptom relief when conventional antidepressant strategies fail. While its pharmacological efficacy has been demonstrated in randomized controlled trials, less attention has been paid to the organizational, relational, and multidisciplinary aspects that influence its real-world implementation and clinical effectiveness. While practical recommendations for intranasal esketamine services exist, an implementation-ready framework integrating psychiatry, nursing, and psychotherapy across treatment phases is still lacking. This narrative review synthesizes the clinical and real-world evidence and proposes a phase-based integration framework with explicit role delineation and measurable implementation/fidelity indicators. **Methods**: We conducted a narrative review informed by a structured literature search in major databases from inception to the most recent update. Search terms combined ‘esketamine’/‘Spravato’ with ‘treatment-resistant depression’, ‘nursing’, ‘psychotherapy’, ‘multidisciplinary’, and ‘implementation’. Outcomes prioritized in the synthesis included depressive symptom severity/response, relapse prevention, safety/tolerability, anhedonia, suicidality monitoring, functional outcomes, and patient-reported experience/retention. Based on this evidence, an integrated, phase-based multidisciplinary framework for esketamine treatment was developed. **Results**: Available evidence supports the efficacy of intranasal esketamine in reducing depressive symptoms in TRD, with growing real-world data confirming its effectiveness and safety. Beyond global symptom improvement, studies highlight benefits on clinically relevant domains such as anhedonia and suicidality trajectories, as well as meaningful patient-reported outcomes. However, the complexity of esketamine delivery requires structured clinical pathways. The proposed model delineates complementary roles for medical supervision, nursing care, and psychotherapy across pre-treatment assessment, induction and session delivery, post-session integration, and maintenance phases, emphasizing safety, continuity of care, and patient-centred monitoring. **Conclusions**: Intranasal esketamine represents not only a pharmacological innovation but also a treatment that necessitates an integrated multidisciplinary approach. A structured phase-based multidisciplinary approach may support safer, more acceptable delivery of intranasal esketamine and potentially improve retention and patient experience; however, prospective implementation and comparative studies are needed to evaluate clinical effectiveness, feasibility, and cost-effectiveness.

## 1. Introduction

Treatment-resistant depression (TRD) represents one of the most complex and burdensome conditions in contemporary psychiatry. Despite the availability of multiple antidepressant classes and psychotherapeutic approaches, a substantial proportion of patients fail to achieve adequate symptom remission after sequential, guideline-concordant treatments. Throughout this manuscript, TRD is operationally defined as inadequate response to at least two antidepressant trials of adequate dose and duration within the current depressive episode [1]. As well as persistent mood symptoms, TRD is associated with chronic functional impairment, a diminished quality of life, an increased risk of medical conditions and sustained use of healthcare resources. This places a considerable burden on patients, their families and the mental health care system [1]. In recent years, the introduction of intranasal esketamine has represented a paradigm shift in the pharmacological management of TRD. By targeting glutamatergic neurotransmission through non-competitive NMDA receptor antagonism, esketamine differs mechanistically from traditional monoaminergic active principles, demonstrating a rapid onset of therapeutic effects in both randomized controlled trials (RCTs) and long-term maintenance studies [2,3,4,5]. However, unlike conventional drugs which are prescribed for unsupervised daily use, esketamine must be administered within a regulated, session-based clinical framework which requires direct supervision and structured monitoring. This distinctive mode of delivery has substantial clinical implications. Esketamine treatment cannot be conceptualized as solely a pharmacological intervention; rather, it is embedded within a therapeutic context that includes the treatment setting, the organization of care, and the coordinated involvement of various professional roles. Consequently, patients’ outcomes may be shaped not only by drug-related factors but also by the manner in which treatment is delivered, monitored, and integrated over time, particularly for session-based interventions requiring repeated in-clinic administration [6,7,8,9,10]. While esketamine’s efficacy and safety have been extensively examined in controlled trials, increasing attention is directed toward its real-world implementation, patient experience, and clinically meaningful outcomes beyond global symptom scores. In parallel, the acute psychoactive effects of esketamine, such as dissociation, perceptual changes, and transient autonomic responses, raise important questions about the role of nursing care and psychological support in ensuring treatment is safe, tolerable, and therapeutically coherent. Although transient psychoactive effects are expected and typically resolve within the observation window, esketamine services must clearly distinguish these from adverse events requiring escalation or discontinuation through standardized monitoring; operational criteria are detailed in Section 3 and Section 5.3.

The aim of this narrative review is twofold. Firstly, we summarize the current evidence on intranasal esketamine for TRD, integrating data from RCTs and real-world studies with a focus on delivery-related factors. Secondly, we propose an integrated therapeutic framework that explicitly incorporates psychiatric oversight, nursing care, and psychotherapeutic support as interdependent components of esketamine treatment. The proposed framework is primarily intended for supervised outpatient/day-hospital esketamine services; core safety and communication principles may be adapted to inpatient contexts where staffing and monitoring resources may differ.

### Methods

This narrative review was informed by a structured literature search and a targeted review of regulatory and clinical guidance documents relevant to intranasal esketamine services. Electronic searches were performed in PubMed/MEDLINE, Embase, PsycINFO, and the Cochrane Library from database inception to 30 November 2025 (last search update: 30 November 2025), restricted to English-language sources. Search terms combined “esketamine” OR “Spravato” with “treatment-resistant depression” OR “TRD”, and implementation-relevant keywords (e.g., “nursing”, “psychotherapy”, “multidisciplinary”, “implementation”, “service model”, “clinic”, “monitoring”, “real-world”, “observational”, and “patient experience”). Because evidence on psychotherapy integration specific to intranasal esketamine remains limited, a supplementary targeted search using “ketamine” combined with “psychotherapy”, “integration”, and “cognitive behavioral therapy” was used as indirect evidence where explicitly indicated.

Eligible sources included RCTs and maintenance studies of intranasal esketamine in adults with TRD, real-world observational studies and post-approval safety analyses, qualitative/patient-experience studies relevant to care delivery, and consensus statements, practical recommendations, and regulatory documents informing monitoring and organizational requirements. Case reports and phenomenological descriptions were included only to inform patient-centred communication and hypothesis generation. Preclinical and pediatric studies were excluded. Titles/abstracts and full texts were screened for relevance by two authors, with disagreements resolved by discussion and consensus. Key information was extracted on study design, population/setting, dosing schedules, main clinical outcomes, and implementation-oriented variables. Given the narrative and framework-development aims, no formal risk-of-bias appraisal tool was applied; evidence was weighted qualitatively by study design and sample size, and recommendations are presented as pragmatic and conditional where direct comparative evidence is lacking.

## 2. Esketamine in Treatment-Resistant Depression: Current Evidence and Open Questions

### 2.1. Evidence from Randomized Controlled Trials

The clinical development programme for intranasal esketamine has provided robust evidence of its antidepressant efficacy in adults with TRD when administered alongside an oral antidepressant. Key design features and principal efficacy and safety findings from pivotal randomized trials and maintenance studies of intranasal esketamine in treatment-resistant depression are summarized in Table 1. Short-term studies have consistently shown relevant reductions in depressive symptom severity during the induction phase compared with placebo-controlled comparators, with treatment effects emerging within days rather than weeks [2,3]. These findings are particularly relevant in populations characterized by long-standing non-response to standard pharmacotherapy. Beyond the initial response, maintenance studies have demonstrated that the continued administration of esketamine to patients who respond to treatment reduced the risk of relapse compared with treatment discontinuation, thus supporting its role as a long-term therapeutic option for selected patients [4]. Importantly, these trials were conducted within tightly structured protocols that included standardized dosing schedules, predefined monitoring procedures, and consistent follow-up. Despite these strengths, randomized trials also leave several questions unresolved. Trial populations are highly selected, monitoring intensity exceeds that in routine care, and outcome measures primarily focus on clinician-rated symptom scales. For example, individuals with substantial medical comorbidity, active substance-use disorders, psychotic features, marked social instability, or other complex presentations are often under-represented in trials yet commonly encountered in routinary clinical practice. Consequently, while the trials have high internal validity, the generalizability of the findings to heterogeneous real-world populations and service settings remains a critical area for further investigation.

### 2.2. Safety Profile and Regulatory Considerations

The safety profile of esketamine has been well characterized in clinical trials and subsequent post-marketing surveillance. Common adverse effects include transient dissociation, dizziness, nausea, sedation and increased blood pressure, which typically occur shortly after administration and resolve within the observation period [3,5]. Serious adverse events are uncommon when treatment is delivered according to regulatory requirements. Therefore, regulatory agencies have mandated that it be administered under supervision in certified healthcare settings, with supervised post-dose monitoring to ensure patient safety. Because monitoring mandates and certification pathways differ across jurisdictions (e.g., U.S. REMS requirements versus nationally defined supervised-use conditions and guidance in Europe/UK), the proposed model should be adapted to local regulatory and governance requirements. These requirements emphasize that esketamine is not just another antidepressant but a treatment that integrates safety procedures into its therapeutic delivery. In this context, staff training and implementation fidelity may be one of the key factors in determining the real-world effectiveness. Operational session-level monitoring requirements (including minimum observation parameters, discharge criteria, and escalation triggers) are detailed in the nursing workflow section and within Phase 2 of the proposed model (Section 3 and Section 5.3).

### 2.3. Real-World Evidence and Clinical Heterogeneity

**Figure 1:**
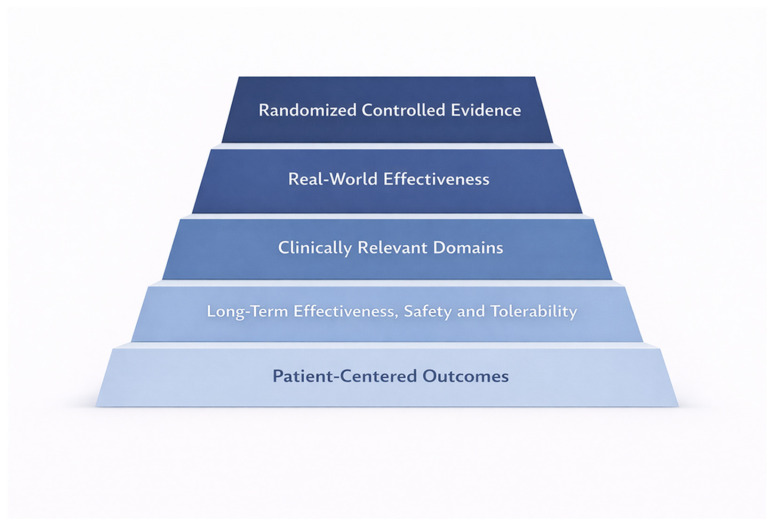
Evidence base supporting intranasal esketamine in treatment-resistant depression (TRD). The pyramid schematically summarizes the main pillars of evidence discussed in this review: randomized controlled evidence, real-world effectiveness, clinically relevant domains, long-term effectiveness/safety/tolerability, and patient-centred outcomes.

The available evidence supporting the use of intranasal esketamine in TRD treatment also encompasses real-world effectiveness, domain-specific clinical outcomes, long-term effectiveness and safety and tolerability data, as well as patient-centred measures. This is summarized schematically in Figure 1. Although RCTs are vital for establishing the efficacy and safety of intranasal esketamine under standardized conditions, real-world studies are crucial for understanding how this intervention performs in routine clinical practice. Such research captures a broader, more heterogeneous patient population, including individuals with long illness durations, multiple prior treatment failures, psychiatric comorbidities and variable adherence patterns. These features are often underrepresented in RCTs. Multicentre real-world cohorts have generally confirmed that intranasal esketamine is associated with meaningful reductions in depressive symptom severity over time and is well tolerated when administered in accordance with regulatory requirements. However, in addition to replicating trial-level outcomes, real-world data has begun to address clinically relevant questions that extend beyond total depression scores. This sheds light on symptom dimensions, patient experience, and risk-related trajectories.

One emerging area of research concerns domain-specific outcomes, particularly anhedonia. In a secondary analysis from the REAL-ESK Study Group (*n* = 253; 199 with unipolar TRD and 54 with bipolar TRD), anhedonia was quantified using the MADRS anhedonia subscale and assessed at baseline and at 1 month and 3 months. Significant improvements in anhedonia were observed over time in both groups (*p* < 0.001), with 38% of unipolar TRD patients and 51.92% of bipolar TRD patients achieving a ≥50% reduction on the anhedonia subscale at 3 months. These data suggest that esketamine may be effective in treating symptoms related to reward that often do not respond well to traditional antidepressant strategies [9]. Another important contribution of real-world research relates to patient-reported experience. The multicentre REAL-ESKperience study examined how patients perceive intranasal esketamine treatment in routine care settings, considering subjective benefits, tolerability and the overall treatment satisfaction. The results showed that patients generally had positive experiences when esketamine was administered in a structured and supportive clinical environment, emphasizing the importance of organizational factors, staff engagement, and continuity of care. These results reinforce the idea that patient experience is a meaningful component of treatment effectiveness, particularly for interventions requiring repeated in-clinic administration [10]. Finally, emerging retrospective and case-based reports—recognizing that intranasal esketamine is not universally approved for an acute suicidality indication—underscore the need for systematic monitoring of suicidality/self-harm risk and for careful clinical framing (and post-session integration) of subjective experiences, particularly in patients with prominent dissociative/depersonalization features; however, these observations are hypothesis-generating and cannot support causal or anti-suicidal effectiveness claims [11,12].

Taken together, real-world evidence portrays intranasal esketamine as a treatment whose clinical impact is shaped by multiple interacting factors: symptom domain specificity, patient experience, risk trajectories, and subjective phenomenology. These data complement randomized trial findings and reinforce the view that esketamine should be evaluated and delivered within integrated care models that attend not only to symptom reduction but also to functional recovery, safety, and experiential dimensions.

## 3. The Role of Nursing Care in Esketamine Treatment Pathways

The administration of intranasal esketamine necessitates a degree of clinical supervision, placing nursing care at the heart of treatment implementation. Unlike standard antidepressant prescriptions, its administration takes place during repeated, time-limited clinical encounters involving pre-dose assessment, supervised self-administration, structured observation and discharge evaluation. Within this framework, nursing staff are responsible for translating regulatory requirements into consistent, therapeutically meaningful clinical practice [13,14]. From a safety perspective, their responsibilities include systematically monitoring physiological parameters and mental status during and after administration, paying particular attention to transient increases in blood pressure, sedation, dissociative phenomena and subjective distress. Large post-approval safety analyses conducted in routine clinical settings have confirmed that the safety profile of intranasal esketamine remains consistent with that observed in RCT. In addition, reports from early clinical experience in routine practice have provided further insights into the feasibility, tolerability and initial management of patients during the implementation of intranasal esketamine outside RCT settings [15]. However, conceptualizing nursing care solely in terms of safety surveillance can lead to an underestimation of its broader clinical role. Nursing care also provides continuity, predictability and relational containment (i.e., structured reassurance, consistent staffing, predictable procedures, and a calm supportive presence), elements that are particularly important for patients with long-standing illness and repeated experiences of treatment failure. Implementation-oriented guidance on esketamine services emphasizes that patient preparation, clarity of procedures and continuity of staff are essential for maintaining engagement across sessions [14,16].

Real-world evidence and qualitative research suggest that patient engagement with and acceptance of esketamine treatment are strongly influenced by the quality of interpersonal interactions and the stability of the care environment. Patients often report heightened vulnerability related to the anticipation of acute psychoactive effects and uncertainty regarding symptom trajectories. In this context, the presence of trained nursing staff who can provide reassurance, clear explanations and emotional support contributes to a sense of safety that goes beyond physiological monitoring [17]. Nursing care also plays a key role in treatment continuity and adherence. As esketamine necessitates frequent clinic visits during the induction phase, logistical challenges, ambivalence or anxiety may arise at the outset of the treatment. Nurses are often the first professionals to detect early warning signs of disengagement, distress or adverse experiences emerging. Recognizing these signals allows for proactive interventions, such as providing additional psychoeducation, adapting the session structure or coordinating with the prescribing psychiatrist, thus preventing premature discontinuation [16]. Crucially, the repeated nature of esketamine sessions fosters an ongoing therapeutic relationship between patients and nursing staff. This enables the longitudinal observation of subtle changes in mood, behaviour and functioning that may not be fully captured by standardized rating scales. Such observational continuity is particularly valuable in real-world settings where symptom trajectories are heterogeneous and influenced by contextual stressors, comorbidities and psychosocial factors.

From an organizational standpoint, nursing care represents the operational backbone of esketamine administration settings. When it comes to service implementation, experts repeatedly emphasize the relevance of standardized protocols, checklists, and staff training programmes. These measures ensure consistency and safety across sessions while allowing for enough flexibility to adapt care to the individual needs of each patient [14,18]. Balancing procedural rigour with clinical responsiveness is essential to preserve the therapeutic dimension of care and avoid a purely technical delivery model. A practical session-level nursing workflow and minimum monitoring/documentation checklist are summarized in Table 2.

Finally, patient-reported experience data from multicentre observational studies indicate that treatment acceptability and perceived benefit are closely linked to staff availability, communication quality, and the environmental features. These findings support the inclusion of nursing-sensitive outcomes, such as perceived safety, clarity of information and relational continuity, as meaningful indicators of service quality in esketamine programmes [10,17].

Overall, nursing care in intranasal esketamine treatment should be considered a therapeutic function rather than a mere procedural requirement. By providing safety, containment, continuity and the early detection of clinically relevant signals, nursing staff contribute directly to the effectiveness and sustainability of esketamine treatment within integrated care models.

## 4. Psychotherapy and Psychological Integration in Esketamine Treatment

In TRD, psychotherapy has demonstrated efficacy as an add-on strategy even after multiple pharmacological failures, supporting its relevance in advanced treatment stages [19,20]. However, the unique pharmacodynamic profile and delivery structure of esketamine necessitate reconfiguring how, when and for what purposes psychological interventions are applied. Integrating psychotherapy into intranasal esketamine treatment requires shifting the conceptual focus from viewing psychotherapy as an adjunctive or parallel intervention to understanding it as a contextual and temporal moderator of treatment outcomes.

### 4.1. Conceptual Rationale for Integration

Esketamine produces rapid changes in mood, cognition, and affective processing that may precede broader functional recovery. These rapid shifts can create a mismatch between symptom improvement and entrenched cognitive, behavioural and interpersonal patterns. From a psychotherapeutic perspective, this temporal dissociation presents both opportunities and risks: while symptom relief may enhance engagement and hope, insufficient integration may limit the durability of change or contribute to confusion and destabilization. Theoretical models of ketamine-assisted psychotherapy propose that acute neurobiological changes may temporarily increase cognitive and emotional flexibility, facilitating subsequent psychological work [20]. Although there is limited direct evidence for structured psychotherapy combined specifically with intranasal esketamine, this framework offers a clinically plausible rationale for integrating psychotherapy as a consolidation mechanism rather than as a simultaneous intervention during the acute effects of the drug.

### 4.2. Timing and Feasibility in Routine Care

Practical constraints play a decisive role in shaping the integration of psychotherapy. Regulatory guidance emphasizes that the immediate post-dose observation period is generally not suitable for formal psychotherapy due to the transient dissociative and sedative effects, as well as the mandatory safety monitoring required [13,21]. This is consistent with practical recommendations for the provision of esketamine services in routine care [14,17]. While regulatory constraints regarding the post-dose observation period are clear, the empirical basis for optimal timing of psychotherapeutic integration with intranasal esketamine is limited, warranting future prospective evaluation. Consequently, integrated models should prioritize delivering psychotherapy outside of dosing sessions, typically before treatment initiation and during the inter-session or maintenance phases. In practice, this creates three feasible intervention windows:

1. Preparation: expectation-setting, psychoeducation, and coping strategy delivery prior to the first esketamine sessions [14,17].
2. Post-session integration: delivered once the acute effects have fully subsided (e.g., after recovery and discharge or on the following day), with the aim of contextualizing subjective experiences and reinforcing adaptive interpretations [16,20,22].
3. Maintenance-oriented psychotherapy: delivered during the continuation/maintenance phases to target behavioural activation, relapse prevention, and functional recovery as treatment progresses [18,19,23].

Although this staging is informed by regulatory and feasibility considerations, direct empirical validation of this specific timing protocol for psychotherapy combined with intranasal esketamine remains absent, representing an important area for prospective investigation. Accordingly, the proposed timeframes should be interpreted as a pragmatic, constraint-derived framework rather than as evidence-derived timing guidance.

### 4.3. Therapeutic Targets and Clinical Orientation

Rather than favouring a single psychotherapeutic approach, integrated esketamine pathways may benefit from a function-oriented approach. Cognitive behavioural strategies, for example, can be useful for consolidating gains through behavioural activation and cognitive restructuring, particularly in areas such as anhedonia and avoidance. Meanwhile, interpersonal and psychodynamic-informed approaches can support meaning-making and relational functioning, and acceptance-based strategies can help patients to tolerate residual symptoms and uncertainty. Evidence from broader ketamine research supports this pluralistic stance. For example, a proof-of-concept randomized trial demonstrated that cognitive behavioural therapy could sustain the antidepressant effects of ketamine infusions in patients with TRD, suggesting a role for psychotherapy in extending the benefits of medication over time [23]. While methodologically heterogeneous, observational studies of ketamine-assisted psychotherapy similarly report symptom improvements and highlight the importance of structured psychological support [20,22]. Although these findings cannot be directly generalized to intranasal esketamine, they do reinforce the clinical plausibility of integration.

### 4.4. Working with Dissociation and Altered Experiences

Dissociative and perceptual changes are among the most distinctive subjective effects of intranasal esketamine. For most patients, these experiences are transient and incidental; they should not be considered therapeutic targets in themselves. Post hoc analyses of pivotal trials indicate that dissociation is neither necessary for, nor correlated with, an antidepressant response, and dose selection should therefore prioritize clinical response and tolerability rather than the elicitation of dissociative symptoms [24]. Overemphasizing dissociation may foster unrealistic expectations or inadvertently encourage inappropriate therapeutic intensity.

When dissociation is distressing, the immediate goals are safety, reassurance, and symptom containment, in close coordination with nursing monitoring. Practical steps include: (i) calm and consistent reassurance and brief orientation (e.g., normalizing the experience and providing time anchors); (ii) minimizing sensory stimulation (e.g., a quiet room with dim lighting and limited interruptions); (iii) simple grounding strategies (paced breathing, brief reality orientation and naming neutral stimuli in the room); and (iv) clear staff communication and documentation of severity and duration by staff. A psychiatric review should be considered when dissociation is accompanied by severe anxiety/panic, agitation, psychotic-like symptoms, emergent suicidality, or prolonged sedation or when symptoms persist beyond the expected observation/recovery period and compromise safe discharge [13]. These considerations underscore the importance of post-session integration (after full recovery) rather than in-session interpretation during the acute effects of the drug.

### 4.5. Interface with Nursing Care and Facility Organization

The effective integration of psychotherapy is inseparable from nursing care and facility organization. Nurses often act as intermediaries between pharmacological treatment and psychological therapy, identifying patients who may benefit from additional support and facilitating communication within the multidisciplinary team. This is particularly important when patients report distressing experiences (e.g., anxiety, dissociation, or feeling overwhelmed) or when their motivation fluctuates during the initial stages of treatment. From a systems perspective, integrating psychotherapy into esketamine services requires explicit coordination rather than informal referral. Clear pathways, shared documentation and regular team communication enhance continuity and reduce fragmentation.

At minimum, shared documentation should include the following: (i) session-level administration details (date/time, dose, concomitant antidepressant, staff present); (ii) an adverse-event log (symptoms, severity, interventions, outcomes); (iii) physiological monitoring (baseline and post-dose blood pressure/heart rate); (iv) mental-status monitoring (sedation and dissociation/distress ratings, if used, grounding/support strategies provided); (v) discharge and safety items (fitness for discharge, driving restrictions and next appointment); and (vi) brief patient-reported experience items (e.g., perceived support, tolerability, and willingness to continue).

A feasible cadence for multidisciplinary reviews is to hold a brief team huddle after each dosing day to flag adverse events, repeated missed visits, emerging risks and clinically significant distress. This should be combined with a formal multidisciplinary review at least weekly during induction and at key decision points (e.g., dose changes, response/non-response decisions and transition to maintenance). During maintenance, reviews can be scheduled every 4–6 weeks, with ad hoc reviews triggered by repeated non-adherence, persistent blood pressure elevations, clinically significant dissociation/sedation or an escalating risk of suicidality. Patient-reported experience data indicate that coherent, well-coordinated care pathways are perceived as more supportive and acceptable, reinforcing the importance of organizational integration [10].

Psychotherapy in intranasal esketamine treatment should be conceptualized neither as an optional add-on nor as a concurrent intervention during acute drug effects. Instead, it functions as a temporal and contextual integrator, supporting preparation, consolidation, and long-term functional recovery. By reconciling theoretical models of rapid-acting antidepressants with practical constraints of routine care, integrated psychotherapeutic approaches can enhance both the effectiveness and the acceptability of the treatment within multidisciplinary frameworks.

## 5. A Proposed Multidisciplinary Model for Esketamine Treatment

**Figure 2:**
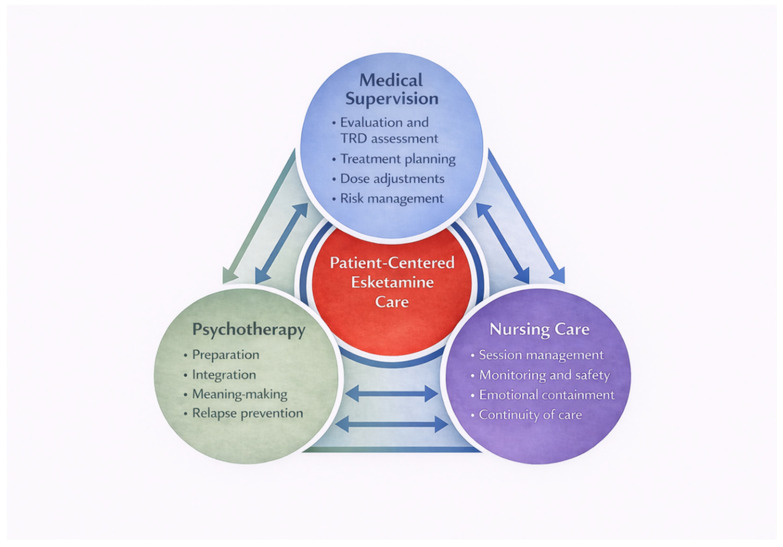
An integrated, multidisciplinary model for intranasal esketamine treatment in patients with treatment-resistant depression (TRD). The diagram illustrates the coordinated responsibilities of medical supervision (including TRD assessment, treatment planning, dose adjustments, and risk management), nursing care (session management, monitoring/safety, emotional support, and continuity of care), and psychotherapy (preparation, post-session integration, meaning-making, and relapse prevention). Bi-directional arrows indicate structured communication and feedback between these roles, with patient-centred esketamine care at the core.

The distinctive pharmacological profile and delivery requirements of intranasal esketamine necessitate a structured clinical model integrating pharmacological efficacy with the organizational, relational and psychological components of care. Drawing on available randomized evidence, real-world data and implementation-oriented literature, we propose a multidisciplinary, phase-based model in which psychiatric governance, nursing care and psychotherapy work together as interdependent elements rather than operating in isolation. Figure 2 schematically represents the proposed model, illustrating the three coordinated roles within a patient-centred esketamine treatment pathway.

### 5.1. Core Principles of the Integrated Model

The proposed model is based on four core principles. First, the context in which esketamine is delivered shapes its clinical impact, as does the continuity of care. Second, roles must be explicit: clarity regarding responsibilities reduces fragmentation and enhances safety. Third, timing is critical, as different professional contributions are most effective at different phases of treatment. Fourth, patient experience and clinically relevant domains should be monitored alongside global symptom severity in order to inform clinical decision-making. In order to make monitoring both feasible and scalable, services may adopt a minimal monitoring set that combines safety indicators (e.g., blood pressure readings, sedation/dissociation/distress monitoring and adverse event logs) with pragmatic outcome indicators such as retention/attendance (e.g., missed visits), brief symptom severity ratings, functional outcomes and short patient-reported experience measures (e.g., perceived support and tolerability). These principles align with international expert guidance on the implementation of esketamine, which emphasizes structured pathways, multidisciplinary coordination and standardized yet flexible protocols [25,26].

### 5.2. Phase 1: Pre-Treatment Assessment and Preparation

The pre-treatment phase is coordinated by the psychiatrist and focuses on confirming the diagnosis, assessing treatment resistance and evaluating medical and psychiatric contraindications. It also includes a measurement-based baseline assessment, paying attention to symptom domains that may influence treatment response and monitoring needs. Nursing staff contribute by reinforcing psychoeducation, clarifying the procedural aspects of treatment and identifying potential logistical or emotional barriers to adherence. When available, psychotherapy is introduced in a preparatory role to support expectation-setting, coping strategies and risk management. This preparatory work is particularly important for patients with high anticipatory anxiety or previous negative treatment experiences.

### 5.3. Phase 2: Induction and Session-Based Treatment Delivery

During the induction phase, esketamine is administered in repeated supervised sessions, in accordance with regulatory and clinical protocols. Nursing care is central to this process, ensuring physiological monitoring, emotional support and consistent procedures throughout the sessions. From an implementation standpoint, the minimum session protocol should include: (i) a pre-dose assessment, including vital signs, particularly blood pressure, and a review of relevant contraindications and recent changes in medical status; (ii) supervised self-administration; (iii) post-dose observation for at least two hours, including a blood pressure measurement around 40 min post-dose and thereafter as clinically indicated, alongside monitoring for sedation, dissociation/distress and other adverse events [13]; (iv) clear discharge criteria, including clinical stability of mental status and vital signs, safe ambulation, and safety advice including not driving or operating machinery until the next day [13]; and (v) predefined escalation triggers to distinguish between expected transient effects and events requiring prolonged observation or urgent medical review (e.g., persistent clinically significant hypertension, prolonged sedation/altered consciousness, severe agitation or psychotic-like symptoms and emergent suicidality) [13,14,17]. These operational elements are consistent with practical recommendations and national guidance for esketamine service delivery [14,17,21]. Psychiatrists are responsible for dose adjustments, clinical decision-making and managing any adverse events. During this phase, psychotherapy does not usually take a structured form on dosing days. Instead, psychological support is coordinated around treatment sessions and focuses on brief check-ins, managing distress and scheduling post-session integration work.

### 5.4. Phase 3: Post-Session Integration and Consolidation

Once the acute effects have resolved, psychotherapy takes on a more active role. Post-session integration focuses on contextualizing subjective experiences, reinforcing adaptive interpretations and translating symptom changes into behavioural and functional improvements. Cognitive behavioural strategies may be useful for addressing avoidance and anhedonia, while acceptance-based and interpersonal approaches can support emotional regulation and relational functioning. Nursing staff continue to contribute by monitoring engagement, identifying emerging concerns and facilitating communication within the team. This phase exemplifies the interface function of nursing care, bridging pharmacological treatment and psychological integration.

### 5.5. Phase 4: Maintenance and Longitudinal Monitoring

**Figure 3:**
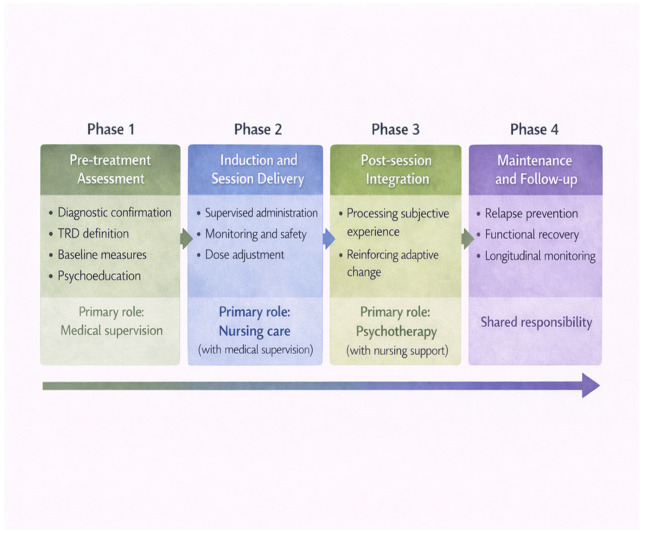
Timing and procedural phases in the proposed integrated intranasal esketamine treatment pathway for treatment-resistant depression (TRD): Phase 1: Pre-treatment assessment. Phase 2: Induction and session delivery. Phase 3: Post-session integration. Phase 4: Maintenance and follow-up. ‘Primary role’ indicates the lead professional’s contribution to each phase, and the horizontal arrow denotes temporal progression and an increase in shared responsibility over time.

The maintenance phase focuses on preventing relapse, promoting functional recovery and assessing ongoing risk. As esketamine administration becomes less frequent, psychotherapy can focus on consolidating progress, managing residual symptoms and re-engaging with social and occupational roles. Real-world evidence supports the relevance of this longitudinal approach. Observational studies have highlighted improvements in domain-specific outcomes, such as anhedonia, and have documented meaningful patient-reported benefits when treatment is delivered within coherent care pathways [9,10]. Furthermore, as data on suicidality-related outcomes remains largely observational, services should prioritize systematic risk assessment and clear escalation pathways throughout treatment, rather than focusing solely on reducing symptoms in the short term. The timing and the procedural phases of the proposed model are schematically summarized in Figure 3.

### 5.6. Organizational Requirements and Scalability

Implementing this integrated model requires organizational investment, particularly during service set-up and the induction phase. In line with practical recommendations and national guidance, key requirements include standardized protocols, staff training, shared documentation systems and scheduled multidisciplinary communication [14,17,21,25]. At the same time, implementation must also allow for local adaptation, given that facility structures, staffing models and patient case mix differ across health systems [14,17,25].

To support scalability, a pragmatic minimum resource checklist should include: (i) a dedicated low-stimulation space for supervised administration and recovery; (ii) basic monitoring equipment consistent with regulatory requirements (e.g., blood pressure device) and fall-risk prevention measures; (iii) access to emergency response procedures and medications according to local policy; (iv) staff competencies in esketamine administration, vital-sign monitoring, recognition/management of sedation and dissociation/distress, and escalating crises; and (v) structured templates for session documentation (dose/time, adverse events, monitoring schedule, discharge criteria, and brief patient-reported experience items) [13,14,17,21,25].

Staffing standards vary by jurisdiction and local governance. As a range of implementation standards are consistent with clinic setup guidance, services may operate with at least one trained nurse present throughout the post-dose observation period for two to three concurrent patients. The prescribing psychiatrist should be immediately available on site (or within the facility) for dose decisions and adverse event management. Higher-risk patients may require one-to-one nursing observation and lower patient throughput, particularly during the initial sessions [13,14,17,21,25].

A key scalability principle is stepped integration intensity, which is based on patient risk and complexity rather than a one-size-fits-all approach. Based on regulatory constraints and expert guidance, Table 3 summarizes a pragmatic risk-stratification matrix that links common clinical indicators to “minimum viable”, “core”, or “full” integrated pathways, with predefined escalation triggers (e.g., repeated missed visits, distressing dissociation/panic, persistent blood pressure elevations, or emerging suicidality risk) [13,14,17,21,25]. Qualitative and patient experience data further highlight that coherent, well-coordinated pathways are perceived as more supportive and acceptable, thus supporting the rationale for explicitly organized escalation and follow-up processes [10,16].

From an economic perspective, integrated pathways are likely to increase demands on staff time and infrastructure. Direct evidence on the incremental cost-utility or budget impact of multidisciplinary integrated care (beyond standard psychiatric monitoring) remains limited. Where available, national appraisals (e.g., NICE) demonstrate how cost-effectiveness considerations interact with service delivery requirements. This highlights the need for future implementation studies to incorporate pragmatic resource indicators and cost-effectiveness/budget impact analyses [21]. Real-world safety analyses also emphasize the importance of robust monitoring and governance as services scale up [27].

### 5.7. Summary

This integrated multidisciplinary model conceptualizes intranasal esketamine as a context-dependent intervention, the outcomes of which are shaped by both pharmacological action and structured clinical care. By aligning psychiatric oversight, nursing expertise, and phase-appropriate psychotherapeutic support across treatment phases, the model aims to enhance safety, continuity of care, patient adherence, and experience while supporting the durability of clinical gains. Future prospective studies should evaluate whether such integrated pathways yield superior outcomes compared to medication-centred delivery alone.

## 6. Limitations

This narrative review and the proposed integrated clinical model have several limitations that must be acknowledged. First, this work is narrative in nature and does not follow a systematic methodology. Although literature was selected to reflect clinically relevant, international and real-world evidence, no formal protocol was applied for identifying studies, appraising their quality or conducting a quantitative synthesis. Consequently, the emphasis placed on specific domains (e.g., nursing care, patient experience, symptom dimensions) reflects clinical relevance and feasibility rather than a predefined evidentiary hierarchy. Second, many of the questions addressed, such as the organization of services, interdisciplinary coordination and the integration of psychotherapy, are primarily informed by observational studies, qualitative research and implementation-oriented guidance. While these sources enhance external validity, they are limited by potential confounding factors, selection bias and variability in clinical practice. Third, the evidence base for esketamine includes several industry-sponsored clinical trials, which may influence the design, reporting and framing of outcomes. In this narrative synthesis, we sought to mitigate this issue by interpreting efficacy findings cautiously and by giving weight to independent post-marketing safety analyses, real-world observational studies and implementation-oriented guidance when discussing service delivery, acceptability and care models. Fourth, evidence supporting the integration of psychotherapy within esketamine pathways remains largely indirect. While psychotherapy has been shown to be effective in treating TRD, and proof-of-concept studies suggest that it plays a part in maintaining the antidepressant effects of ketamine, there is a lack of controlled trials that specifically evaluate the combination of structured psychotherapeutic interventions and intranasal esketamine. Consequently, the proposed psychotherapeutic framework should be considered theoretically grounded and clinically plausible but not yet standardized. Fifth, the real-world data incorporated into this review mainly derive from retrospective or naturalistic designs. While these studies provide valuable insights into routine care, they cannot establish causality or fully disentangle the effects of treatment from contextual and patient-related factors. Sixth, the proposed integrated model assumes the availability of trained nursing staff, coordinated multidisciplinary teams and dedicated infrastructure. These resources may not be accessible everywhere, which could limit the model’s generalisability and scalability, particularly in under-resourced settings. Finally, the manuscript does not provide a validated implementation toolkit, such as standardized operating procedures, competency frameworks or audited quality indicators. The proposed model is therefore intended as a conceptual and clinical template that requires local adaptation, governance oversight and prospective evaluation before it can be adopted as a standard approach to care.

## 7. Conclusions

The evidence discussed in this manuscript supports the view that intranasal esketamine is a context-dependent intervention whose effectiveness and acceptability are influenced by how it is delivered and monitored over time. Nursing care plays a central role in providing safety, continuity and structured support during repeated sessions. Psychotherapy, meanwhile, can contribute by preparing patients, facilitating post-session integration and consolidating functional recovery. Real-world studies further reinforce the need for outcome frameworks that extend beyond symptom severity to include pragmatic functional outcomes and patient-reported experience. Overall, these considerations support the rationale for integrated, multidisciplinary care pathways that align psychiatric oversight, nursing expertise, and psychological support across treatment phases while enabling stepped implementation based on local resources and patient needs. Future prospective and implementation-focused studies should evaluate whether integrated models improve long-term outcomes compared to medication-centred delivery alone and examine patient preferences regarding the intensity and timing of psychotherapeutic support.

## Figures and Tables

**Table 1:** Key pivotal trials of intranasal esketamine in treatment-resistant depression (selected).

Study	Design/Population	Intervention	Comparator	Primary Endpoint	Key Results
TRANSFORM-1 (Fedgchin 2019) [5]	Phase 3 RCT; outpatient. Adults with TRD (nonresponse ≥ 2 antidepressants). N = 346.	ESK NS 56 or 84 mg BIW x4w + new OAD.	Placebo NS BIW x4w + new OAD.	Change in MADRS (Day 28).	84 mg vs. placebo: LSMD −3.2; *p* = 0.088 (primary endpoint not met). 56 mg: nominal LSMD −4.1; nominal *p* = 0.027 (not formally tested by hierarchy).
TRANSFORM-2 (Popova 2019) [2]	Phase 3 RCT; outpatient. Adults with TRD. N = 227 randomized.	Flex-dose ESK NS 56/84 mg BIW x4w + new OAD.	Placebo NS BIW x4w + new OAD.	Change in MADRS (Day 28; MMRM).	Day 28: LSMD −4.0 (95% CI −7.31 to −0.64); *p* = 0.020. Discontinued due to AEs: 7.0% vs. 0.9%.
SUSTAIN-1 (Daly 2019) [4]	Randomized withdrawal maintenance. After 16w open-label ESK + OAD. N = 297 entered randomized maintenance.	Continue ESK NS (56/84 mg; weekly or q2w) + OAD.	Switch to placebo NS + OAD.	Time to relapse (stable remission primary).	Relapse: stable remission 26.7% vs. 45.3% (HR 0.49; *p* = 0.003). Stable response 25.8% vs. 57.6% (HR 0.30; *p* < 0.001).

Abbreviations: AE, adverse event(s); BIW, twice weekly; CI, confidence interval; ESK NS, esketamine nasal spray; HR, hazard ratio; LSMD, least squares mean difference; MADRS, Montgomery–Åsberg Depression Rating Scale; MMRM, mixed model for repeated measures; NS, nasal spray; OAD, oral antidepressant; q2w, every 2 weeks; RCT, randomized controlled trial; TRD, treatment-resistant depression; w, weeks.

**Table 2:** Example nursing workflow and monitoring checklist for intranasal esketamine sessions.

Session Stage	Key Nursing Actions	Minimum Monitoring and Documentation	Escalation/Safety Notes
Pre-session (T0)	Confirm indication/eligibility; review recent medical changes and key contraindications; orient patient to expected transient effects; prepare a low-stimulation environment with fall-risk precautions	Record date/time, dose plan; baseline BP/HR; brief mental-status	Seek medical review if baseline BP is markedly elevated per local protocol, if there is acute intoxication/withdrawal, emergent suicidality/psychosis/mania, or other clinical instability
Administration	Supervise self-administration; ensure safe posture (seated/reclined) and direct observation; maintain a calm and predictable session structure	Document time of dosing, administered dose, staff present; note any immediate adverse effects and supportive measures provided	Escalate if acute agitation, severe anxiety/panic, chest pain, syncope, or other concerning symptoms occur during/soon after administration
Post-dose observation (0–2 h minimum)	Monitor for sedation, dissociation/distress, nausea/dizziness; provide reassurance and grounding as needed; maintain fall-risk precautions and minimize stimulation	Repeat BP/HR around ~40 min post-dose and thereafter as clinically indicated until values decrease; document sedation/dissociation/distress (scale if used, otherwise structured checklist); record AE, interventions, and resolution	Prolong observation and request psychiatric/medical review for persistent clinically significant hypertension, prolonged sedation/altered consciousness, severe agitation or psychotic-like symptoms, respiratory concerns, or emergent suicidality
Discharge and follow-up	Confirm clinical stability; ensure safe ambulation and orientation; provide post-visit advice; schedule next session	Document discharge criteria met (vital signs stable, mental status, adverse events resolved); reinforce no driving/operating machinery until the next day; record follow-up	Delay discharge if the patient is not clinically stable; initiate escalation pathway if safety concerns persist

Abbreviations:; AE, adverse event; BP, blood pressure; HR, heart rate.

**Table 3:** Suggested risk stratification for tailoring integration intensity in intranasal esketamine services.

Risk Stratum	Pragmatic Indicators (Examples)	Recommended Integration Intensity	Operational Notes
Lower clinical risk/complexity	Stable baseline functioning; no active suicidality; low anxiety; good adherence and support; no clinically significant dissociation/sedation; reliable transport/home supervision.	Minimum viable integrated pathway	Standard psychiatric oversight + nursing safety monitoring; psychoeducation and brief check-ins; escalation triggers (e.g., AE, rising risk, repeated non-adherence) prompt step-up to higher-intensity integration.
Moderate clinical risk/complexity	No acute suicidality; moderate symptom burden or functional impairment; manageable anxiety; mild–moderate dissociation; stable housing/support; generally adherent.	Core integrated pathway	Standard safety-focused nursing monitoring; brief preparation session(s) and targeted integration sessions; MDT review weekly during induction then every 2–4 weeks in maintenance.
Higher clinical risk/complexity	Active suicidal ideation or recent self-harm; severe anticipatory anxiety; prominent dissociation/panic during sessions; complex trauma or severe comorbidity; repeated missed visits or poor social support.	Full integrated pathway	Prefer 1:1 nursing observation during dosing; structured pre-treatment psychotherapy preparation; scheduled post-session integration (same day after recovery or within 24–72 h); MDT review at least weekly during induction and as needed.

Note: This matrix is intended as a pragmatic, implementation-oriented framework (not a validated tool). Local regulatory requirements, service capacity, and individual clinical judgement should guide final decisions. Abbreviations: AE, adverse event; MDT, multidisciplinary team.

## Data Availability

No new datasets were generated or analyzed in this review.

## Abbreviations

The following abbreviations are used in this manuscript:

TRD	Treatment-resistant depression
NMDA	N-methyl-D-aspartate
RCT	Randomized controlled trials
